# Supplementary material for: Impact of long COVID on health-related quality-of-life: an OpenSAFELY population cohort study using patient-reported outcome measures (OpenPROMPT)
Source: Lancet Reg Health Eur. 2024 Apr 24;40:100908. doi: 10.1016/j.lanepe.2024.100908 (PMC11059448; doi:10.1016/j.lanepe.2024.100908)
Supplement: Supplementary File 1 [file mmc1.docx]

**Appendix: The Impact of Long COVID on HRQoL using OpenPROMPT**

# Supplementary methods

## Information governance and ethical approval

This research is part of the OpenPROMPT study “Quality-of-life in patients with long COVID: harnessing the scale of big data to quantify the health and economic costs” which has ethical approval from HRA and Health and Care Research Wales (HCRW) (IRAS project ID 304354). The Study Coordination Centre has obtained approval from the LSHTM Research Ethics Committee (ref 28030), as well as a favourable opinion from the South Central—Berkshire B Research Ethics Committee (ref 22/SC/0198).

LSHTM is the data controller of OpenPROMPT data. NHS England is the data controller of the NHS England OpenSAFELY COVID-19 Service. TPP is the data processor; all study authors using OpenSAFELY have the approval of NHS England (1). This implementation of OpenSAFELY is hosted within the TPP environment which is accredited to the ISO 27001 information security standard and is NHS IG Toolkit compliant (2).

Patient data has been pseudonymised for analysis and linkage using industry standard cryptographic hashing techniques; all pseudonymised datasets transmitted for linkage onto OpenSAFELY are encrypted; access to the NHS England OpenSAFELY COVID-19 service is via a virtual private network (VPN) connection; the researchers hold contracts with NHS England and only access the platform to initiate database queries and statistical models; all database activity is logged; only aggregate statistical outputs leave the platform environment following best practice for anonymisation of results such as statistical disclosure control for low cell counts (3).

The service adheres to the obligations of the UK General Data Protection Regulation (UK GDPR) and the Data Protection Act 2018. The service previously operated under notices initially issued in February 2020 by the Secretary of State under Regulation 3(4) of the Health Service (Control of Patient Information) Regulations 2002 (COPI Regulations), which required organisations to process confidential patient information for COVID-19 purposes; this set aside the requirement for patient consent (4). As of 1 July 2023, the Secretary of State has requested that NHS England continue to operate the Service under the COVID-19 Directions 2020 (5). In some cases of data sharing, the common law duty of confidence is met using, for example, patient consent or support from the Health Research Authority Confidentiality Advisory Group(6).

Taken together, these provide the legal bases to link patient datasets using the service. GP practices, which provide access to the primary care data, are required to share relevant health information to support the public health response to the pandemic, and have been informed of how the service operates.

## OpenPROMPT

OpenPROMPT was supported by the National Institute for Health and Care Research (NIHR) as a collaboration between the London School of Hygiene and Tropical Medicine, the OpenSAFELY data platform at the University of Oxford, and The Phoenix Partnership (TPP), which supplies software to General Practices in the UK. Any adult in England could participate in the study, provided they were able to download and use the Airmid smartphone application. Participants could join either passively, discovering the study page in the Airmid Research Module, or through active discovery from Airmid push notifications which invited them towards the OpenPROMPT study page. General Practices using TPP SystmOne software were invited to participate in disseminating the study to patients in their system. The study was also shared on social media to promote both the general population and individuals highly impacted by long COVID to enrol.

## Long COVID Histories in Electronic Health Records (EHRs)

Alongside the questionnaire data collection, histories related to COVID-19 in medical records utilised codes created in OpenSAFELY. For the comparison between the numbers self-reporting long COVID to diagnosed cases in EHRs, definition of a long COVID diagnosis was based on SNOMED CT codes from NICE guidelines on managing the long-term effects of COVID-19:

| **SNOMED CT Code** |  |
| --- | --- |
| 1325161000000102 | Post-COVID-19 syndrome |
| 1325181000000106 | Ongoing symptomatic disease caused by severe acute respiratory syndrome coronavirus 2 |

For wider assessment of long COVID histories, the assessment of any record relating to long COVID was based on codes including referral to specialist long COVID clinics:

**SNOMED CT Code**

| 1325021000000106 | Signposting to Your COVID Recovery |
| --- | --- |
| 1325031000000108 | Referral to post-COVID assessment clinic |
| 1325041000000104 | Referral to Your COVID Recovery rehabilitation platform |
| 1325051000000101 | Newcastle post-COVID syndrome Follow-up Screening Questionnaire |
| 1325061000000103 | Assessment using Newcastle post-COVID syndrome Follow-up Screening Questionnaire |
| 1325071000000105 | COVID-19 Yorkshire Rehabilitation Screening tool |
| 1325081000000107 | Assessment using COVID-19 Yorkshire Rehabilitation Screening tool |
| 1325091000000109 | Post-COVID-19 Functional Status Scale patient self-report |
| 1325101000000101 | Assessment using Post-COVID-19 Functional Status Scale patient self-report |
| 1325121000000105 | Post-COVID-19 Functional Status Scale patient self-report final scale grade |
| 1325131000000107 | Post-COVID-19 Functional Status Scale structured interview final scale grade |
| 1325141000000103 | Assessment using Post-COVID-19 Functional Status Scale structured interview |
| 1325151000000100 | Post-COVID-19 Functional Status Scale structured interview |

Hospitalisation with COVID-19 was defined using data from Hospital Episode Statistics Admitted Patient Care (HES-APC) accessed through OpenSAFELY. The dataset used ICD-10 codes to define any previous COVID-19 related hospitalisation.Though U072 states that COVID-19 was not identified, the code was used when suspected but testing proved inconclusive:

| **icd10_code** |  |
| --- | --- |
| U071 | covid19 virus identified |
| U072 | covid19 virus not identified |

## Supplementary Methods References

1. NHS Digital. NHS Digital. [cited 2023 Nov 17]. The NHS England OpenSAFELY COVID-19 service - privacy notice. Available from: https://digital.nhs.uk/coronavirus/coronavirus-covid-19-response-information-governance-hub/the-nhs-england-opensafely-covid-19-service-privacy-notice

2. NHS Digital. NHS Digital. [cited 2023 Nov 17]. Data Security and Protection Toolkit. Available from: https://digital.nhs.uk/data-and-information/looking-after-information/data-security-and-information-governance/data-security-and-protection-toolkit

3. NHS Digital. NHS Digital. [cited 2023 Nov 17]. ISB1523: Anonymisation Standard for Publishing Health and Social Care Data. Available from: https://digital.nhs.uk/data-and-information/information-standards/information-standards-and-data-collections-including-extractions/publications-and-notifications/standards-and-collections/isb1523-anonymisation-standard-for-publishing-health-and-social-care-data

4. GOV.UK. GOV.UK. 2022 [cited 2023 Nov 17]. Coronavirus (COVID-19): notice under regulation 3(4) of the Health Service (Control of Patient Information) Regulations 2002 – general. Available from: https://www.gov.uk/government/publications/coronavirus-covid-19-notification-of-data-controllers-to-share-information/coronavirus-covid-19-notice-under-regulation-34-of-the-health-service-control-of-patient-information-regulations-2002-general--2

5. NHS Digital. NHS Digital. [cited 2023 Nov 17]. COVID-19 Public Health Directions 2020. Available from: https://digital.nhs.uk/about-nhs-digital/corporate-information-and-documents/directions-and-data-provision-notices/secretary-of-state-directions/covid-19-public-health-directions-2020

6. NHS HRA. Health Research Authority. [cited 2023 Nov 17]. Confidentiality Advisory Group. Available from: https://www.hra.nhs.uk/about-us/committees-and-services/confidentiality-advisory-group/

# Supplementary figures


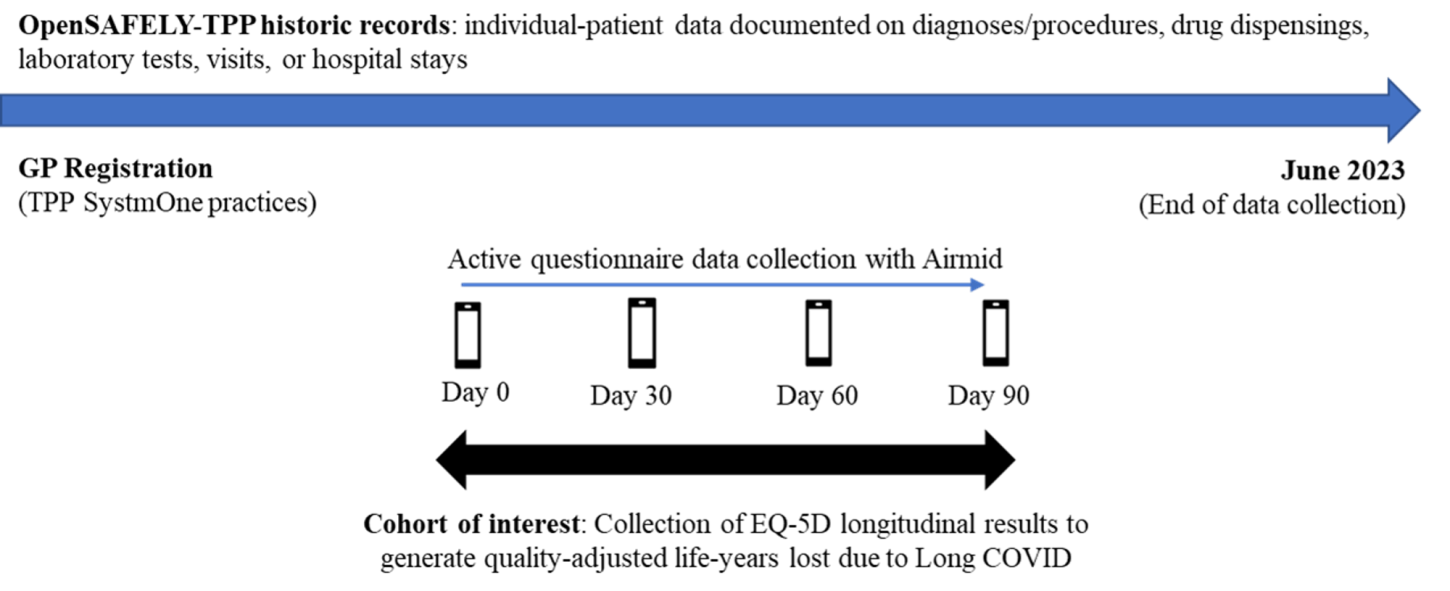


**Supplementary figure 1.** Study design diagram of the primary cohort of OpenPROMPT.


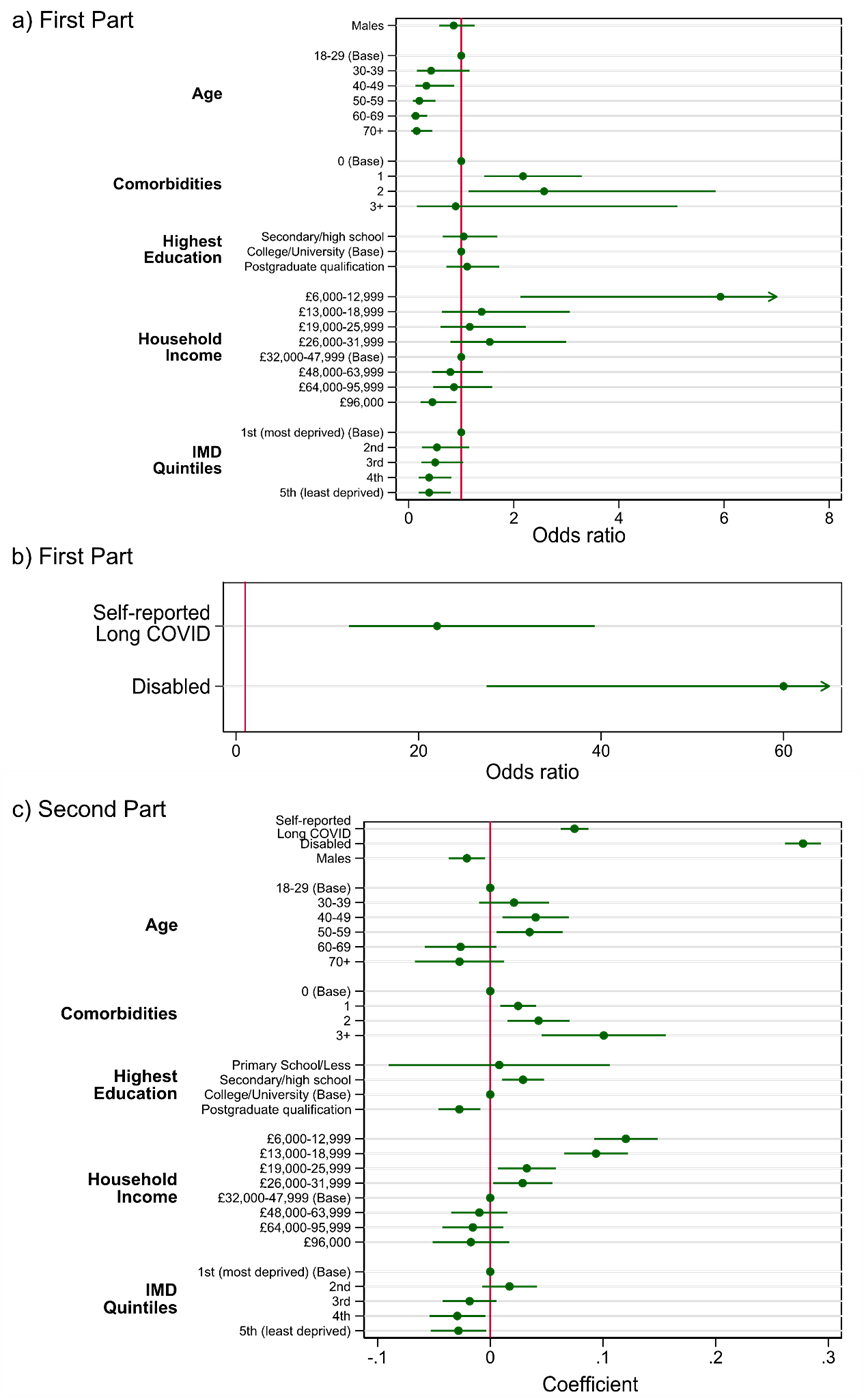


**Supplementary Figure 2. Model outputs for disutility using no imputed data**. a) Odds ratios for the probability of reporting disutility in the first part of the full model. Note that greater odds ratio relates to a higher odds of reporting a negative change in HRQoL. b) Odds ratios for self-reported long COVID and disability in the first part of the model shown separately to allow visualisation, due to their much higher odds ratios. c) Coefficients for the second part of the model, interpreted as the unit decrease in EQ-5D-5L utility score compared to base level for factor variables for individuals who report loss of HRQoL. Note that negative coefficients relate to lower disutility, i.e. higher quality-of-life.


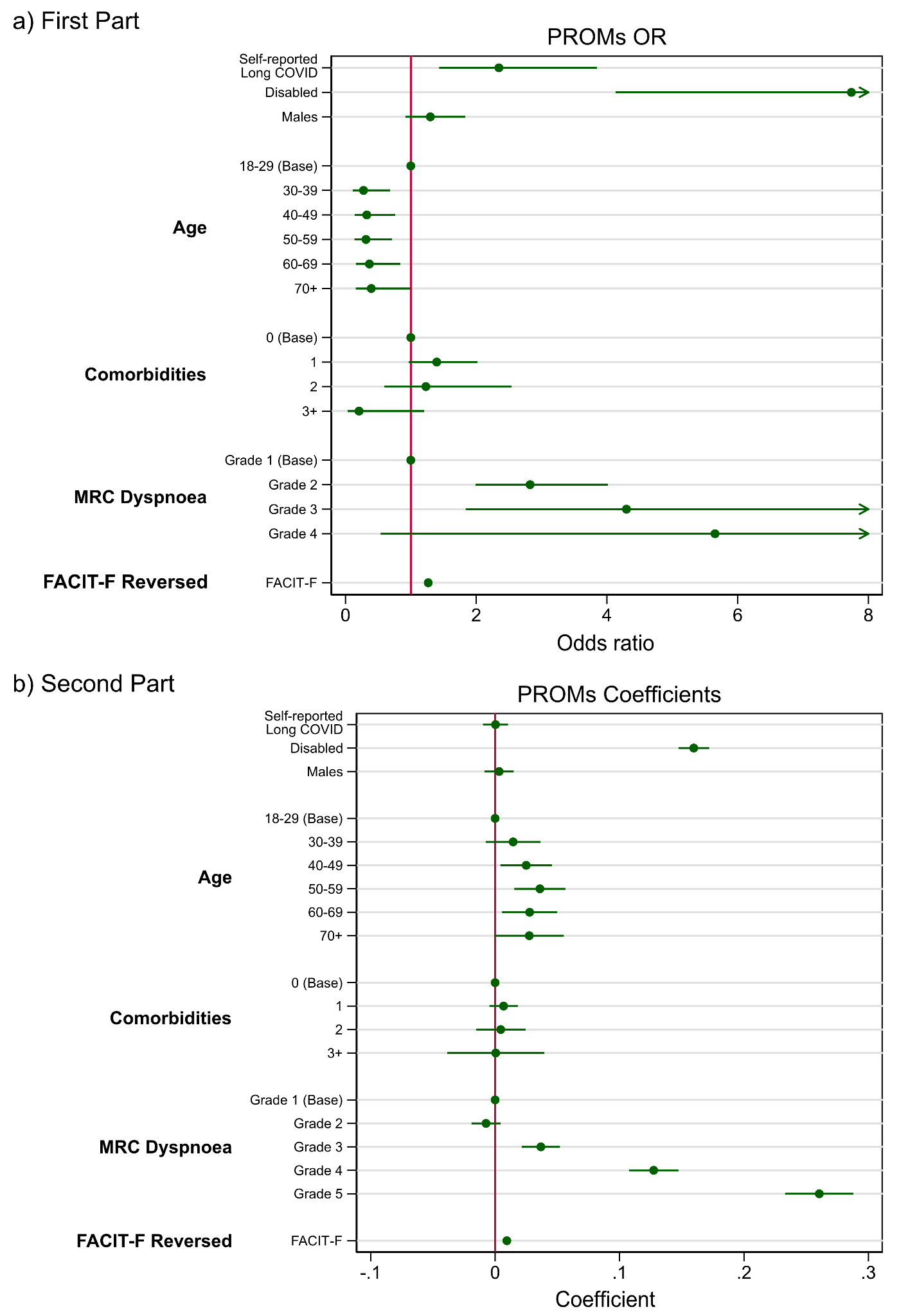


**Supplementary Figure 3. Model outputs for disutility including additional Patient-Reported Outcome Measures (PROMS) using no imputed data.** a) Odds ratios for the probability of reporting disutility in the first part of the full model including PROMs. Note that greater odds ratio relates to a higher odds of reporting a negative change in disutility. b) Coefficients for the second part of the model include PROMs, interpreted as the unit decrease in EQ-5D-5L utility score compared to base level for factor variables for individuals who report loss of HRQoL. Note that negative coefficients relate to lower disutility, i.e. higher quality-of-life.


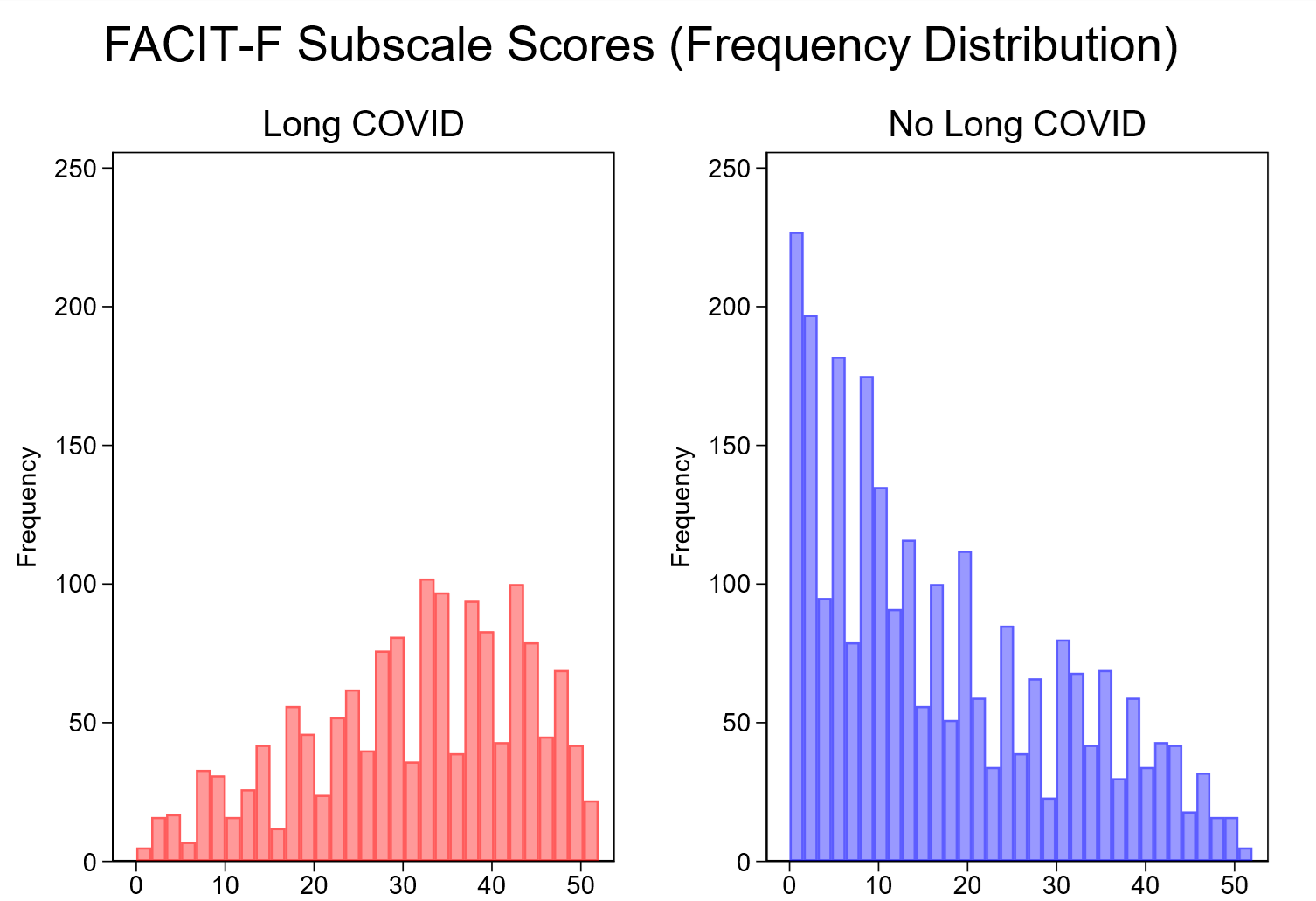


**Supplementary figure 4.** Frequency distribution of reversed FACIT-F fatigue scores at recruitment survey stratified by self-reported long COVID. Higher scores indicate greater fatigue


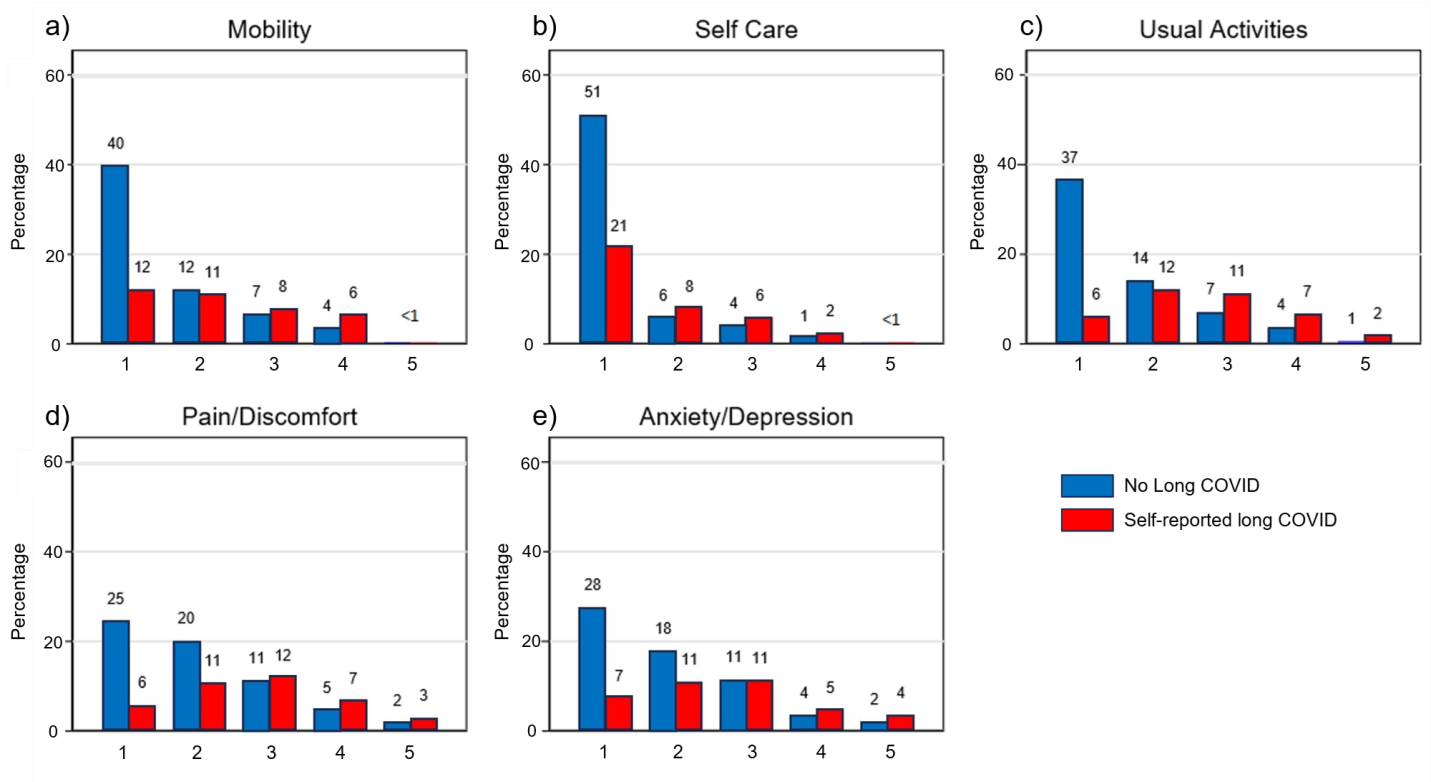


**Supplementary figure 5.** Self-reported quality-of-life measures. Percentage responses for the five dimensions of HRQoL measured in EQ-5D-5L. a) Mobility dimension of ED-5D, b) Self-care dimension of EQ-5D, c) Usual activities dimension of ED-5D. d) Pain/discomfort dimension of ED-5D, e) Anxiety/depression dimension of ED-5D. Each dimension has five possible responses: level 1: no problems, level 2: slight problems, level 3: moderate problems, level 4: severe problems, and level 5: extreme problems/unable to. Blue marks the participant did not report long COVID, and red that they did. Responses are only shown for the 3975 non-missing self-reported long COVID respondents

# Supplementary tables

## Supplementary table 1. List of Comorbidities

| **Disease** |
| --- |
| Non-haematological cancer |
| Haematological cancer^1^ |
| Chronic respiratory disease |
| Chronic cardiac disease |
| Chronic liver disease |
| Stroke or dementia |
| Other neurological conditions^2^ |
| Mental health conditions^3^ |
| Organ transplant |
| Rheumatoid arthritis |
| Systemic lupus erythematosus |
| Psoriasis |
| Other immunosuppressive conditions^4^ |

1. Having haematological cancers six months before the index date; 2. Such as Huntington’s disease, multiple sclerosis, motor neuron diseases, and other neurological diseases; 3. Consists of psychosis, schizophrenia, bipolar disorder, and depression; 4. Including other permanent and temporary immunosuppressive diseases.

|  | **First Part**  **Probability of HRQoL loss** | | **Second Part**  **Unit loss in HRQoL** | |
| --- | --- | --- | --- | --- |
|  | **Odds ratio** | **95% CI** | **Coefficients** | **95% CI** |
|  |  | | | |
| **Self reported long COVID** | 4·70*** | [3·72, 5·93] | 0·056*** | [0·04, 0·07] |
| **Disabled** | 17·7*** | [10·37, 30·33] | 0·26*** | [0·24, 0·28] |
| **Males** | 0·76* | [0·59, 0·98] | -0·024*** | [-0·04, -0·01] |
| **Age (Base 18-29)** |  | | | |
| 30-39 | 0·65 | [0·33, 1·26] | 0·019 | [-0·01, 0·05] |
| 40-49 | 0·53 | [0·26, 1·07] | 0·023* | [0·00, 0·04] |
| 50-59 | 0·37* | [0·17, 0·81] | 0·013 | [-0·01, 0·04] |
| 60-69 | 0·26* | [0·1, 0·67] | -0·027* | [-0·05, 0·00] |
| 70+ | 0·24** | [0·1, 0·58] | -0·042** | [-0·07, -0·01] |
| **Comorbidities (Base 0)** |  | | | |
| 1 | 1·55** | [1·21, 1·99] | 0·025*** | [0·01, 0·04] |
| 2 | 1·88** | [1·22, 2·92] | 0·047*** | [0·03, 0·07] |
| 3+ | 2·29 | [0·79, 6·64] | 0·082*** | [0·04, 0·12] |
| **Highest Education (Base college/university)** |  | | | |
| Primary School/Less | 4·08 | [0·08, 207·21] | 0·093 | [0·00, 0·19] |
| Secondary/high school | 1·05 | [0·8, 1·37] | 0·024** | [0·01, 0·04] |
| Postgraduate qualification | 0·8 | [0·63, 1·02] | -0·017* | [-0·03, 0·00] |
| **Household Income (Base £32-000-47999)** |  | | | |
| £6000-12999 | 2·63** | [1·51, 4·59] | 0·11*** | [0·08, 0·13] |
| £13000-18999 | 1·65* | [1·03, 2·64] | 0·075*** | [0·05, 0·10] |
| £19000-25999 | 1·39 | [0·94, 2·05] | 0·040*** | [0·02, 0·06] |
| £26000-31999 | 1·41 | [0·92, 2·15] | 0·028* | [0·00, 0·05] |
| £48000-63999 | 0·8 | [0·6, 1·08] | -0·014 | [-0·03, 0·01] |
| £64000-95999 | 0·76 | [0·52, 1·11] | -0·02 | [-0·04, 0·00] |
| £96000 + | 0·58* | [0·37, 0·91] | -0·025 | [-0·05, 0·00] |
| **IMD Quintiles (Base 1st (most deprived))** |  | | | |
| 2nd | 0·85 | [0·53, 1·37] | -0·012 | [-0·03, 0·01] |
| 3rd | 0·75 | [0·47, 1·20] | -0·024** | [-0·04, -0·01] |
| 4th | 0·62 | [0·34, 1·11] | -0·042*** | [-0·06, -0·02] |
| 5th (least deprived) | 0·59 | [0·31, 1·13] | -0·044*** | [-0·07, -0·02] |

**Supplementary table 2.** Multivariable Mixed effect regression models on the probability of HRQoL loss, and the resulting unit loss associated with covariates using multiple imputation by chained equations (Note: negative coefficients indicate better HRQoL). The log of variance was 4·19*** (95% CI: 3·34, 5·26). The number of observations was 24,276 for the first part and 20,427 for the second part. * p<0·05, ** p<0·01, *** p<0·001

|  | **First Part**  **Probability of HRQoL loss** | | **Second Part**  **Unit loss in HRQoL** | |
| --- | --- | --- | --- | --- |
|  | **Odds ratio** | **95% CI** | **Coefficients** | **95% CI** |
|  |  | | | |
| **Self Reported long COVID** | 1.46 | [0.89, 2.38] | -0.00086 | [-0.02, 0.02] |
| **Disabled** | 4.86*** | [3.61, 6.54] | 0.13*** | [0.12, 0.14] |
| **Males** | 1 | [0.89, 1.25] | -0.0033 | [-0.01, 0.01] |
| **Age (base 18-29)** |  | | | |
| 30-39 | 0.7 | [0.4, 1.23] | 0.012 | [-0.01, 0.03] |
| 40-49 | 0.7 | [0.37, 1.30] | 0.024* | [0.00, 0.04] |
| 50-59 | 0.71 | [0.39, 1.30] | 0.036*** | [0.02, 0.05] |
| 60-69 | 0.79 | [0.5, 1.26] | 0.042** | [0.01, 0.07] |
| 70+ | 0.9 | [0.42, 1.95] | 0.047* | [0.01, 0.08] |
| **Comorbidities (base 0)** |  | | | |
| 1 | 1.17 | [0.83, 1.66] | 0.0032 | [-0.01, 0.02] |
| 2 | 1.36 | [0.76, 2.42] | 0.009 | [-0.01, 0.03] |
| 3+ | 0.95 | [0.28, 3.21] | 0.0069 | [-0.05, 0.06] |
| **Breathlessness scale (base grade 1)** |  | | | |
| MRC Breathlessness Scale: grade 2 | 1.53*** | [1.32, 1.77] | 0.0077 | [0.00, 0.02] |
| MRC Breathlessness Scale: grade 3 | 2.31* | [1.25, 4.24] | 0.055** | [0.03, 0.08] |
| MRC Breathlessness Scale: grade 4 | 4.64 | [0.92, 23.47] | 0.14*** | [0.1, 0.17] |
| MRC Breathlessness Scale: grade 5 | 2.91 | [0.41, 20.87] | 0.24*** | [0.19, 0.30] |
| **FACIT F-score** | 1.19*** | [1.17, 1.22] | 0.0099*** | [0.01, 0.01] |

**Supplementary table 3.** Multivariable Mixed effect regression models on the probability of HRQoL loss with the inclusion of symptom specific patient-reported outcome measures (PROMs), and the resulting unit loss associated with covariates using multiple imputation by chained equations (Note: negative coefficients indicate better HRQoL). The log of variance was 2·92*** (95% CI: 2·34; 3·63). The number of observations was 24,276 for the first part and 20,427 for the second part. * p<0·05, ** p<0·01, *** p<0·001

|  | **First Part**  **Probability of HRQoL loss** | | **Second Part Unit loss in HRQoL** | |
| --- | --- | --- | --- | --- |
|  | **Odds ratio** | **95% CI** | **Coefficients** | **95% CI** |
|  |  | |  | |
| **Self-Reported long COVID** | 21·2*** | [12·03, 37·54] | 0·075*** | [0·06, 0·09] |
| **Males** | 0·9 | [0·61, 1·31] | -0·021* | [-0·04, 0·00] |
| **Age (base 18-29)** |  | |  | |
| 30-39 | 0·39 | [0·14, 1·08] | 0·027 | [0·00, 0·06] |
| 40-49 | 0·31* | [0·12, 0·81] | 0·046** | [0·02, 0·08] |
| 50-59 | 0·19*** | [0·07, 0·48] | 0·039* | [0·01, 0·07] |
| 60-69 | 0·12*** | [0·05, 0·33] | -0·023 | [-0·05, 0·01] |
| 70+ | 0·14*** | [0·05, 0·42] | -0·023 | [-0·06, 0·02] |
| **Number of Comorbidities (Base 0)** |  | |  | |
| 1 | 2·18*** | [1·45, 3·29] | 0·023** | [0·01, 0·04] |
| 2 | 2·85* | [1·25, 6·49] | 0·042** | [0·01, 0·07] |
| 3+ | 0·90 | [0·16, 5·14] | 0·10*** | [0·05, 0·16] |
| **Disabled** | 60·2*** | [27·79, 130·57] | 0·28*** | [0·26, 0·30] |
| **Highest Education (Base College/University)** |  | |  | |
| Primary School/Less | 1 | [1·00, 1·00] | 0·051 | [-0·04, 0·15] |
| Secondary/high school | 1·05 | [0·66, 1·68] | 0·029** | [0·01, 0·05] |
| Postgraduate qualification | 1·14 | [0·74, 1·76] | -0·026** | [-0·04, -0·01] |
| **Household Income (Base £32000-47999)** |  | |  | |
| £6000-12999 | 5·84*** | [2·1, 16·27] | 0·12*** | [0·09, 0·15] |
| £13000-18999 | 1·47 | [0·67, 3·24] | 0·095*** | [0·07, 0·12] |
| £19000-25999 | 1·26 | [0·66, 2·40] | 0·038** | [0·01, 0·06] |
| £26000-31999 | 1·64 | [0·85, 3·18] | 0·027* | [0·00, 0·05] |
| £48000-63999 | 0·81 | [0·46, 1·43] | -0·011 | [-0·04, 0·01] |
| £64000-95999 | 0·87 | [0·48, 1·59] | -0·015 | [-0·04, 0·01] |
| £96000 + | 0·44* | [0·22, 0·89] | -0·017 | [-0·05, 0·02] |
| **IMD Quintiles (Base 1st (most deprived))** |  | |  | |
| 2nd | 0·52 | [0·25, 1·10] | 0·014 | [-0·01, 0·04] |
| 3rd | 0·51 | [0·25, 1·04] | -0·02 | [-0·04, 0·00] |
| 4th | 0·39* | [0·19, 0·81] | -0·03* | [-0·05, -0·01] |
| 5th (least deprived) | 0·40* | [0·19, 0·81] | -0·029* | [-0·05, 0·00] |

**Supplementary table 4.** Multivariable Mixed effect regression models on the probability of HRQoL loss, and the resulting unit loss associated with covariates using no imputed data (Note: negative coefficients indicate better HRQoL). The log of variance was 7·77*** (95% CI: 5·52-10·94). The number of observations was 5253 for the first part, and 4483 for the second part. Akaike Information Criterion (AIC) for the first part was 3240.9 and 2452.3 for the second part. * p<0.05, ** p<0.01, *** p<0.001

Disabled participants had the greatest probability of reporting an impact on quality-of-life (OR for returning a loss of HRQoL: 60·2 (95% CI: 27·79,130·57), and an estimated 0·28 (CI: 0·26; 0·30) unit lower quality-of-life (supplementary table 2). Those with comorbidities reported an impact on quality-oflife, especially evident for one comorbidity (OR:2·18, CI:1·45, 3·29) and decreasing as comorbidities increase. The unit decrease in quality-of-life was significant for all levels in the second part, between 0·023-0·1. No effect was found on the impact of ethnicity across models. Evidence was found on the probability of lower ages reporting worsened quality-of-life**.** Lower quintiles of deprivation and higher levels of household income were found to improve quality-of-life compared to baseline levels. No evidence was found on the probability of education level predicting changes in quality-of-life, but there was a small impact of lower levels having higher unit decreases in quality-of-life.

|  | **First Part**  **Probability of HRQoL loss** | | **Second Part Unit loss of HRQoL** | |
| --- | --- | --- | --- | --- |
|  | **Odds ratio** | **95% CI** | **Coefficients** | **95% CI** |
| **Self reported long COVID** | 2·35*** | [1·37, 3·61] | 0·00093 | [-0·01, 0·01] |
| **Disabled** | 7·49*** | [4·04, 13·89] | 0·16*** | [0·15, 0·17] |
| **Male** | 1·3 | [0·93, 1·83] | 0·0027 | [-0·01, 0·01] |
| **Age (Base 18-29)** |  | |  | |
| 30-39 | 0·26** | [0·11, 0·65] | 0·018 | [0·00, 0·04] |
| 40-49 | 0·31** | [0·13, 0·71] | 0·028** | [0·01, 0·05] |
| 50-59 | 0·31** | [0·14, 0·70] | 0·038*** | [0·02, 0·06] |
| 60-69 | 0·35* | [0·15, 0·80] | 0·030** | [0·01, 0·05] |
| 70+ | 0·37* | [0·15, 0·92] | 0·029* | [0·00, 0·06] |
| **Number of Comorbidities (Base 0)** |  | |  | |
| 1 | 1·45* | [1·01, 2·08] | 0·0067 | [0·00, 0·02] |
| 2 | 1·32 | [0·65, 2·71] | 0·0044 | [-0·02, 0·02] |
| 3+ | 0·23 | [0·04, 1·26] | 0·005 | [-0·03, 0·04] |
| **MRC Breathlessness (Base grade 1)** |  | |  | |
| Grade 2 | 2·89*** | [2·04, 4·08] | -0·0077 | [-0·02, 0·00] |
| Grade 3 | 5·27*** | [2·22, 12·48] | 0·037*** | [0·02, 0·05] |
| Grade 4 | 5·65 | [0·57, 56·40] | 0·13*** | [0·11, 0·15] |
| Grade 5 | 1 | [1·00, 1·00] | 0·26*** | [0·23, 0·29] |
| **FACIT-F score** | 1·26*** | [1·22, 1·30] | 0·0094*** | [0·01, 0·01] |

**Supplementary table 5.** Multivariable Mixed effect regression models on the probability of HRQoL loss with the inclusion of symptom specific patient-reported outcome measures (PROMs), and the resulting unit loss associated with covariates using no imputed data (Note: negative coefficients indicate better HRQoL). The log of variance was 5·20*** (95% CI: 3·52-7·69). The number of observations was 6181 for the first part, and 5433 for the second part. Akaike Information Criterion (AIC) for the first part was 3112·8 and -4850·7 for the second part. * p<0.05, ** p<0.01, *** p<0.001

|  | **QALYs** | |
| --- | --- | --- |
|  | **Long COVID** | **No Long COVID** |
|  | **Mean (SD)** | |
| **1 Month** | 0·043 (0·022) | 0·027 (0·022) |
| **2 Months** | 0·047 (0·024) | 0·025 (0·02) |
| **3 Months** | 0·046 (0·026) | 0·022 (0·02) |
| **Total** | 0·135 (0·068) | 0·073 (0·061) |

**Supplementary table 6.** Individual level Quality-Adjusted life-years (QALYs) stratified by long COVID for complete cases, calculated using Area Under the Curve (AUC). These are reshaped from the QualityAdjusted life-months (QALMs) to reflect life-years.
