# Supplementary material for: Impact of long COVID on health-related quality-of-life: an OpenSAFELY population cohort study using patient-reported outcome measures (OpenPROMPT)
Source: Lancet Reg Health Eur. 2024 Apr 24;40:100908. doi: 10.1016/j.lanepe.2024.100908 (PMC11059448; doi:10.1016/j.lanepe.2024.100908)
Supplement: Supplementary File 2 [file mmc2.docx]

Participant Information Sheet and Consent

**Title of Project: OpenPROMPT: Quality-of-life in patients with and without long COVID**

We would like to invite you to take part in a research study. Joining the study is entirely up to you. Before you decide, you need to understand why the research is being done and what it involves. **Please read this information sheet** and if you have any questions, or if anything is unclear, please contact the study team using the email provided. Please feel free to talk to others about the study if you wish. Take time to decide whether or not to take part.

**What is the study about?**

This study is looking at the impact of long COVID on health-related quality of life. This includes things like symptoms, physical and mental health, and productivity at work. Collecting this information will help us to understand:

- which groups of patients are impacted most by long COVID;
- the impact of long COVID on costs to the NHS and the economy;
- which strategies might help to reduce the chance of people getting long COVID in the future.

**Who is organising and funding this study?**

The London School of Hygiene and Tropical Medicine (LSHTM) and the University of Oxford are organising the study. Both institutions have been carrying out research into COVID-19 since the pandemic started.

LSHTM is the sponsor for the research and has full responsibility for the project including the collection, storage and analysis of your data. LSHTM and the University of Oxford are joint data controllers for the study, which means that they are both responsible for looking after your information and using it properly. The study is funded by the National Institute for Health and Social Care Research (NIHR), which is part of the NHS.

**Why have I been invited to take part?**

The study is inviting any adult in England, including people *with and without* long COVID, to take part.

**Do I have to take part?**

No. It is up to you to decide whether or not to take part.

**What will happen to me if I take part?**

We (the study team at LSHTM) will ask you to complete four sets of short questionnaires using the Airmid app. You will be asked to complete the first set when you opt-in to the study, and then another set at 30 days,

60 days and 90 days afterwards. The set of questionnaires should take less than 15 minutes to complete. The questionnaires will ask about you and your experience of COVID and long COVID (if any), how you rate your health and quality of life, and your experiences of breathlessness, fatigue and productivity.

You will be sent electronic reminders to complete the questionnaires, and you can complete them through the Airmid app whenever you have time.

After 90 days we won’t ask you to do anything else and your active participation in the study will end.

**Can I take part if I am already taking part in another research study?**

Yes, if you are already enrolled in another research study you can also take part in this one. Similarly, if you wish to take part in another research study after joining this one you can do so.

**What are the possible risks and disadvantages?**

There are no risks to taking part, and the only disadvantage to you might be the time taken each month to complete the questionnaires. There are various controls in place regarding how your data is stored and accessed that mean the researchers will not be able to identify you (for example, your name, address, date of birth) in the data that is made available to them, or the results they generate.

**What are the possible benefits?**

We cannot promise any direct benefit to you personally. The information that we get from the study will help our knowledge and understanding of research into long COVID. We hope that this will improve ways of helping people who are at risk of long COVID, and our understanding of long COVID symptoms.

**What if something goes wrong?**

If you have a concern about any aspect of this study, you can email the researchers ([openprompt@lshtm.ac.uk](mailto:openprompt@lshtm.ac.uk)). If you are not satisfied by the response, or wish to complain, you can do this by contacting: Patricia Henley at [rgio@lshtm.ac.uk](mailto:rgio@lshtm.ac.uk) or 020 7927 2626.

The London School of Hygiene and Tropical Medicine holds insurance policies which apply to this study. If you experience harm or injury as a result of taking part in this study, you may be eligible to claim compensation.

**What will happen to information collected about me?**

The questionnaire data that you give us will be stored securely at TPP (a GP surgery software provider, which also provides the Airmid app).

LSHTM will also need to use information from your medical records for this research project. For example, we have commissioned NHS England to link the answers you give in the questionnaires to records that it holds in its OpenSAFELY Coronavirus (COVID-19) Research Platform. These records include information extracted from your routinely held NHS electronic health records, for example, those held by your GP practice, hospitals you have attended and other organisations that record health information (such as COVID tests, COVID vaccinations and death records).

With your consent LSHTM will disclose your questionnaires to NHS England so that they can provide this service for us. You can find out about NHS England’s OpenSAFELY Research Platform here: <https://www.england.nhs.uk/contact-us/privacy-notice/how-we-use-your-information/covid-19>- response/coronavirus-covid-19-research-platform/, and NHS England’s privacy notice is here: <https://www.england.nhs.uk/contact-us/privacy-notice>/

The data in your questionnaires is de-identified by a process called ‘pseudonymisation’ before it is uploaded to OpenSAFELY. This involves removing or replacing any information that might identify you directly. A number that is unique to you, derived from your NHS Number is added to allow for linkage to the other data in the OpenSAFELY database. This is an important security measure to maintain your privacy. Researchers who use the data will NOT be able to see your personal information such as your name, address or date of birth.

At the end of the project, approved researchers will be able to interrogate the linked data in OpenSAFELY in anonymous form. We will write our reports in a way that no-one can work out that you took part in the study.

**What are your choices about how your information is used?**

- You can stop being part of the study at any time, without giving a reason, but we will keep information about you that we already have.
- If you choose to stop taking part in the study, we would like to continue collecting information about your health from NHS records. If you do not want this to happen, tell us and we will stop.
- We need to manage your records in specific ways for the research to be reliable. This means that we won’t be able to let you see or change the data we hold about you.

**Where can you find out more about how your information is used?**

You can find out more about how we use your information

- at [www.hra.nhs.uk/information-about-patients](http://www.hra.nhs.uk/information-about-patients)/, or [www.hra.nhs.uk/patientdataandresearch](http://www.hra.nhs.uk/patientdataandresearch),
- by sending an email to [openprompt@lshtm.ac.uk](mailto:openprompt@lshtm.ac.uk), or
- at [https://www.opensafely.org](https://www.opensafely.org/)/.

**How do I withdraw from the study?**

You can withdraw through the Airmid app by sliding the ‘opt-in’ slider to ‘opt-out’. This will not affect your care and we will not contact you about the study again.

**Will I be reimbursed or receive any payment for participating in this study?**

You will not receive any payment or reimbursement for participating in this study.

**Who has reviewed this study?**

All research involving human participants is looked at by an independent group of people, called a Research Ethics Committee, to protect your interests. This study has been reviewed and given favourable opinion by The London School of Hygiene and Tropical Medicine Research Ethics Committee (TBC). The Proportionate Review Sub-Committee of the South Central - Berkshire B Research Ethics Committee has also reviewed the study and have agreed that we can ask people to take part (REC reference 22/SC/0198).

Approval does not guarantee that you will not come to any harm if you take part. However, approval means that the committee is satisfied that your rights will be respected, that any risks have been reduced to a minimum and balanced against possible benefits and that you have been given sufficient information on which to make an informed decision.

The study has also been reviewed by the NIHR’s Long Covid panel.

**Further information and contact details**

Thank you for taking time to read this information sheet. If you would like any further information or have any questions, please contact the study team: [openprompt@lshtm.ac.uk](mailto:openprompt@lshtm.ac.uk).

Please see the consent statements overleaf

**Your consent to take part in OpenPROMPT**

| **By opting-in to the OpenPROMPT study, you agree to all of the following statements:** |
| --- |
| **Information about the study has been provided to me:**  I have read the information sheet for this study dated 6 July 2022, version 1.3. I understand the information and have had the opportunity to ask questions for clarification. These have been answered satisfactorily. |
| **Voluntary participation:**  I understand that my participation is voluntary and that I am free to withdraw from the study at any time. I do not need to give a reason and my medical care or rights will be unaffected. I can withdraw by moving the ‘opt-in’ toggle to ‘opt-out’. |
| **Access to my information:**  I agree that data collected from me in this study will be linked to routinely held NHS data, for example from my GP, hospitalisation, COVID test, COVID vaccination and death records. An example of where this linked data may be held is by NHS England in the OpenSAFELY database. I understand that information that identifies me will be passed securely to such bodies to make this possible. |
| **Transfer of personal data:**  I understand and agree that to facilitate this research project, the study team may need to share my personal information with other organisations. An example of this will be passing my NHS number and other details to NHS Digital to allow my records to be located and shared with the researchers. |
| **Data regulation:**  I understand that my de-identified and pseudonymised data collected during the study may be looked at by regulatory authorities, authorised individuals from the London School of Hygiene and Tropical Medicine, University of Oxford, funding bodies, regulatory authorities or public health agencies, where it is relevant to my taking part in this research. I give permission for these individuals to have access to my de-identified and pseudonymised records. |
| I understand that the questionnaire data that can identify me will be stored securely at TPP (a GP surgery software provider that also provides the Airmid app). I understand that when this questionnaire data is linked to my other NHS data, this combined data will be stored and controlled in a secure research environment, which provides access to only approved researchers. |
| **Use by other researchers:**  I agree that my research data from the study may be made available to other approved researchers for future ethically approved research. I understand that such researchers will not be able to identify me from my data. |
| **I agree to take part in the above study.** |
| **Participant declaration: I acknowledge that by opting in to the OpenPROMPT study that I agree to all the statements above.**  **I understand that opting in to this study is the equivalent of signing a physical document.** |

*Information sheet and consent statements v1.3 6 July 2022*

Demographic questionnaire

| We would like to ask you for some information about yourself. You can choose ‘prefer not to say’ if you do not want to answer any question. |
| --- |

| What year were you born? |
| --- |
| What is your ethnicity?  *must provide value   1. White – English / Welsh/ Scottish / Northern Irish / British 2. White – Irish 3. White – Gypsy or Irish Traveller 4. White – any other background 5. Mixed/ multiple ethnic backgrounds – White and Black Caribbean 6. Mixed/ multiple ethnic backgrounds – White and Black African 7. Mixed/ multiple ethnic backgrounds – White and Asian 8. Mixed/ multiple ethnic backgrounds - any other mixed/ multiple ethic background 9. Asian/ Asian British – Indian 10. Asian / Asian British – Pakistani 11. Asian / Asian British – Bangladeshi 12. Asian / Asian British – Chinese 13. Asian / Asian British – any other Asian background 14. Black/ African/ Caribbean/ Black British – African 15. Black/ African/ Caribbean/ Black British – Caribbean 16. Black/ African/ Caribbean/ Black British – any other Black/ African/ Caribbean background 17. Other ethnic group – Arab 18. Other ethnic group – any other ethnic group 19. Prefer not to say   (18) Other ethnic group - Any other ethnic group Please describe  * must provide value |
| What is the highest level of education you completed?  *must provide value   1. None 2. Primary School 3. Secondary school (GCSE level, O-level, NVQ level 1/2 or equivalent, typically age 16) 4. Sixth form college (A-levels, NVQ level 3 or equivalent, typically age 18) 5. Vocational qualification (NVQ level 4 or equivalent) 6. Undergraduate university degree or NVQ level 5 or equivalent 7. Post-graduate qualification 8. Prefer not to say |
|  |
| Do you have a disability? (A disability is a physical or mental impairment which impacts on your ability to do day-to-day activities and, which has lasted, or is expected to last for at least 12 months.) *must provide value |

| 1. Yes 2. No 3. Prefer not to say   If yes, how would you describe your disability? * must provide value |
| --- |
| What is your current relationship status?  *must provide value   1. Cohabiting 2. In a civil partnership 3. Married 4. Single (never married or never in a civil partnership) 5. Separated (but still legally married or in a civil partnership) 6. Divorced or civil partnership dissolved 7. Widowed or surviving partner from a civil partnership 8. Prefer not to say |
| What is your gender?  *must provide value   1. Male 2. Female 3. Intersex 4. Non Binary 5. If you prefer to use your own term, please describe below 6. Prefer not to say   (5) Please describe  * must provide value |
| What is your yearly household income (select one)  *must provide value   1. < £19,000 2. £19,001- £26,000 3. £26,001 - £35,000 4. £35,001 - £48,000 5. > £48,001 6. Prefer not to say |
| Please tell us the first part of your postcode |
| Where did you hear about the study? (select one)   1. push notification from Airmid 2. text message from my GP 3. email from my GP 4. facebook 5. twitter 6. Instagram 7. from a friend/family member 8. Other (please state)   8) Must provide value |

END OF QUESTIONNAIRE

Dyspnoea (UKRI. MRC breathlessness scales: 1952 and 1959. Published January 20, 2016. Accessed November 26, 2023. https://www.ukri.org/publications/mrc-breathlessness-scales-1952-and-1959/)

## 1959 MRC Breathlessness Scale

Grade 1: Are you ever troubled by breathlessness except on strenuous exertion?

Grade 2: (If yes) Are you short of breath when hurrying on the level or walking up a slight hill?

Grade 3: (If yes) Do you have to walk slower than most people on the level? Do you have to stop after a mile or so (or after 1⁄4 hour) on the level at your own pace?

Grade 4: (If yes to either) Do you have to stop for breath after walking about 100 yds. (or after a few minutes) on the level?

Grade 5: (If yes) Are you too breathless to leave the house, or breathless after undressing?

END OF QUESTIONNAIRE

Fatigue (FACIT. FACIT-Fatigue. FACIT Group. Accessed November 26, 2023. https://www.facit.org/measures/facit-fatigue)


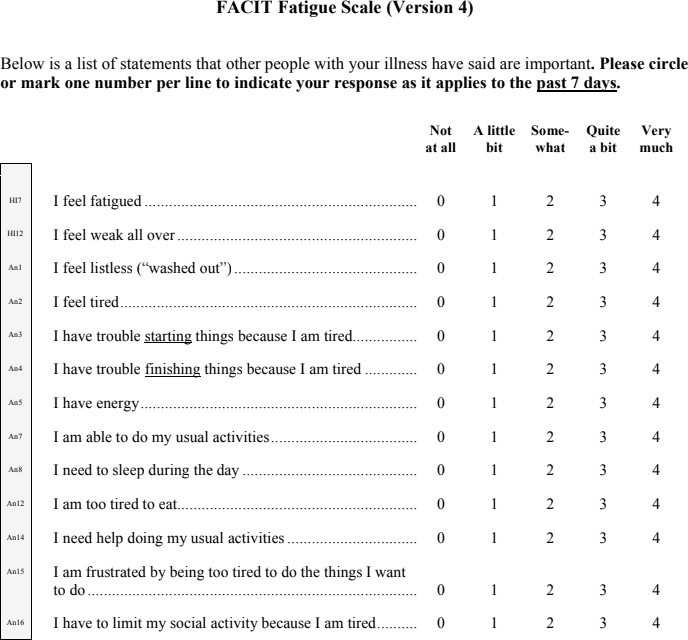


END OF QUESTIONNAIRE

Health-related quality of life (EQ-5D-5L. EuroQol. Accessed November 26, 2023. https://euroqol.org/information-and-support/euroqol-instruments/eq-5d-5l/)


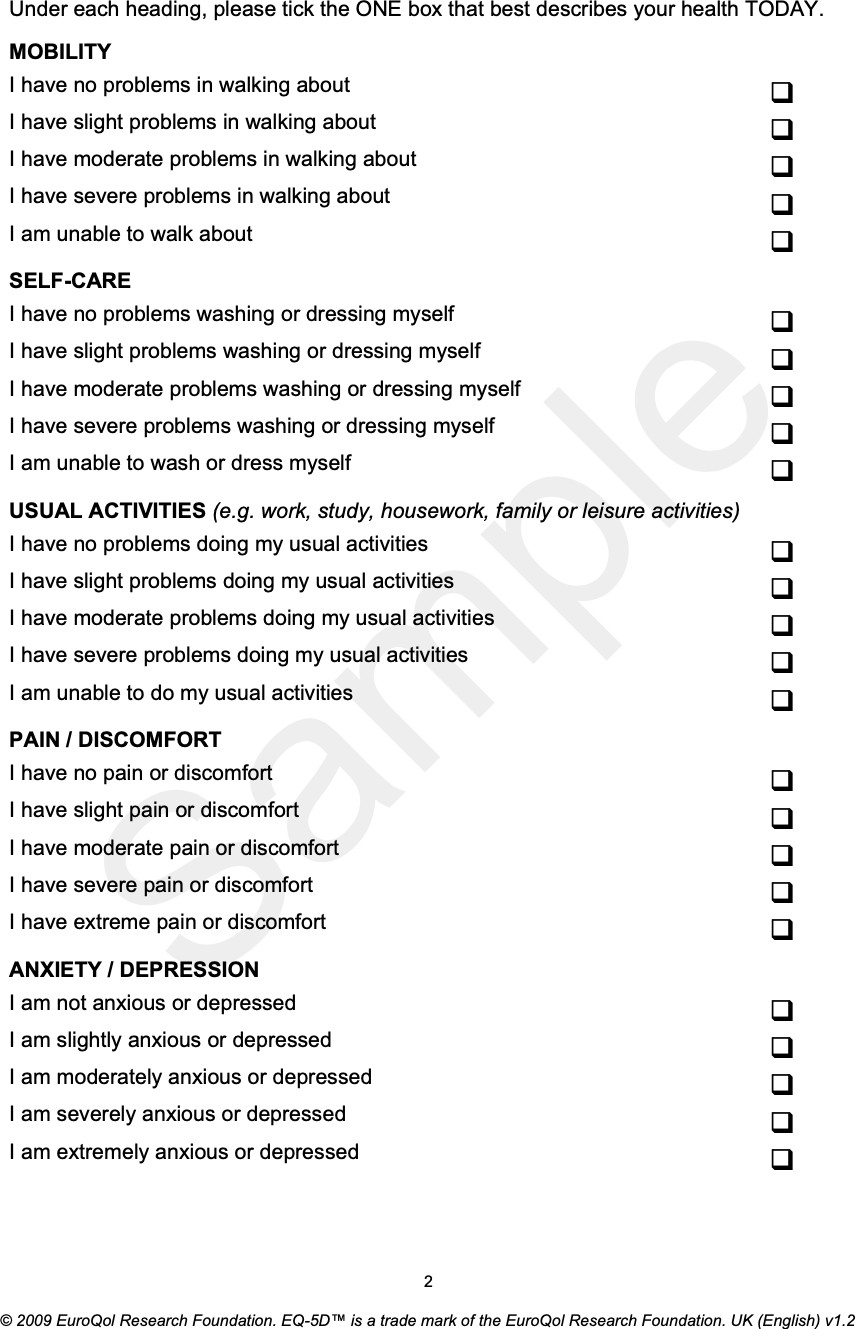


END OF QUESTIONNAIRE

**Work Productivity and Activity Impairment Questionnaire: Specific Health Problem V2.0 (WPAI:SHP)**

(Reilly Associates. WPAI:SHP V2.0. Accessed November 26, 2023. http://www.reillyassociates.net/WPAI_SHP.html)

The following questions ask about the effect of your PROBLEM on your ability to work and perform regular activities. *Please fill in the blanks or circle a number, as indicated.*

1. Are you currently employed (working for pay)? NO YES

*If NO, check “NO” and skip to question 6.*

The next questions are about the **past seven days**, not including today.

1. During the past seven days, how many hours did you miss from work because of problems associated with your PROBLEM? *Include hours you missed on sick days, times you went in late, left early, etc., because of your PROBLEM. Do not include time you missed to participate in this study.*

HOURS

1. During the past seven days, how many hours did you miss from work because of any other reason, such as vacation, holidays, time off to participate in this study?

HOURS

1. During the past seven days, how many hours did you actually work?

HOURS *(If “0”, skip to question 6.)*

1. During the past seven days, how much did your PROBLEM affect your productivity while you were working?

*Think about days you were limited in the amount or kind of work you could do, days you accomplished less than you would like, or days you could not do your work as carefully as usual. If PROBLEM affected your work only a little, choose a low number. Choose a high number if PROBLEM affected your work a great deal.*

Consider only how much PROBLEM affected productivity while you were working.

PROBLEM had no effect on my work

0 1 2 3 4 5 6 7 8 9 10

CIRCLE A NUMBER

PROBLEM completely prevented me from working

1. During the past seven days, how much did your PROBLEM affect your ability to do your regular daily activities, other than work at a job?

*By regular activities, we mean the usual activities you do, such as work around the house, shopping, childcare, exercising, studying, etc. Think about times you were limited in the amount or kind of activities you could do and times you accomplished less than you would like. If PROBLEM affected your activities only a little, choose a low number. Choose a high number if PROBLEM affected your activities a great deal.*

Consider only how much PROBLEM affected your ability to do your regular daily activities, other than work at a job.

PROBLEM had no effect on my daily activities

WPAI:SHP V2.0 (US English)

END OF QUESTIONNAIRE

0 1 2 3 4 5 6 7 8 9 10

CIRCLE A NUMBER

PROBLEM completely prevented me from doing my daily activities

- - 1. COVID, long COVID and vaccination

## Do you think that you currently have or have ever had COVID-19?

- - - - 1. Yes, confirmed by a positive test
        2. Yes, based on medical advice
        3. Yes, based on strong personal suspicion
        4. Unsure
        5. No
        6. Prefer not to say

## When do you think you first got (or might have got) COVID-19? If you do not remember exactly, please put your best estimate.

- - - - 1. MM/YYYY
        2. Don’t know
        3. Prefer not to answer

## How many times do you think you have had an episode of COVID-19?

[enter number]

## Thinking of your last, or only, episode of COVID-19, have you now recovered to normal?

- - - - 1. Yes, I am back to normal
        2. No, I still have some or all of my symptoms
      1. **How long have you had / did you have COVID-19 symptoms overall? Please include time spent with mild symptoms and the time in between symptoms if these have been coming and going.** If you have caught COVID-19 more than once, please answer about the longest episode of illness you experience**.**
         1. Less than 2 weeks
         2. 2 – 3 weeks
         3. 4 – 12 weeks
         4. More than 12 weeks

## Have you had at least one COVID-19 vaccine injection?

- - - - 1. Yes
        2. No – but I intend to
        3. No – and I do not intend to
        4. Don’t know
        5. Prefer not to say

If you have your vaccine card, please use that to help answer the following questions.

## How many COVID-19 vaccine injections have you had?

1 2 3 4 5

- - - 1. **When was your first COVID-19 vaccine injection?** If you can’t remember exactly, please put your best estimate.
         1. (DD / MM / YYYY) _ _ / _ _ / _ _ _ _
         2. Don’t know
      2. **When was your most recent COVID-19 vaccine injection?** If you can’t remember exactly, please put your best estimate.

1. (DD / MM / YYYY) _ _ / _ _ / _ _ _ _
2. Don’t know
